# A systematic review: Investigation of effectiveness of the web based online interventions to manage and reduce stress of university students

**DOI:** 10.1192/j.eurpsy.2021.2004

**Published:** 2021-08-13

**Authors:** A. Ozogul

**Affiliations:** Care Management, United Medical LLC, Ankara, Turkey

**Keywords:** stress management, university students, online interventions, web based interventions

## Abstract

**Introduction:**

The literature shows a high prevalence of psychopathological problems, anxiety and depression among university students because of academic expectations, uncertain future plans, staying away from their family, economic issues and peer relationships. Although these problems show high prevalence among university students, providing them a professional care is limited so most of problems remain untreated. Nowadays the students use digital technologies commonly therefore web based and computer delivered interventions may be useful for them to improve resilience and coping strategies.

**Objectives:**

The intent of the study was to review systematically the impacts of web based and computer delivered interventions regarding stress management among university students.

**Methods:**

Several databases were searched with using key words such as university students, online interventions, web based interventions and stress management. Randomised controlled studies were reviewed.

**Results:**

We found 284 article with the key words. Only four of them met the including criterias. All results of reviewed articles show that web based online interventions have an impact to reduce depression, stress and anxiety level among students. According to the results students improved coping skills against stress after web based online sessions.

**Conclusions:**

The findings show that web based and computed delivered interventions can be effective to improve resilience and reduce students` depression, anxiety and stress symptoms when compared non-interactive and inactive controls. In addition online interventions regarding stress management may provide us to reach out large group of university students.

**Disclosure:**

No significant relationships.

